# Remission effect of Canagliflozin in patients with newly diagnosed type 2 diabetes mellitus: a protocol for a multicenter, parallel-group, randomized, controlled, open-label trial

**DOI:** 10.1186/s12902-023-01461-9

**Published:** 2023-10-10

**Authors:** Xue Yang, Zhiwei He, Li Yuan, Wenbin Huang, Doudou Li, Pingping Xiang, Yu Chen, Guofang Chen, Chao Liu

**Affiliations:** Department of Endocrinology, Affiliated Hospital of Integrated Traditional Chinese and Western Medicine, Nanjing University of Chinese Medicine, Nanjing, China

**Keywords:** Canagliflozin, Type 2 diabetes mellitus, Remission, Metformin, Sodium-glucose cotransporter-2 inhibitor

## Abstract

**Background:**

Studies reporting the effects of metabolic surgery, lifestyle intervention, and intensive insulin therapy for the remission of type 2 diabetes (T2DM) has been increasing, with fruitful results better conducted and yielded. However, there are only a few studies on the remission of T2DM using oral hypoglycemic drugs. Therefore, this study aims to investigate the remission effect of canagliflozin and metformin on participants with newly diagnosed T2DM and its possible underlying mechanism(s) through which these two medications elicit diabetes remission.

**Method:**

To this end, we performed a multicenter, parallel-group, randomized, controlled, and open-label trial. A total of 184 participants with a ≤ 3-year course of T2DM will be enrolled and randomly assigned to the canagliflozin or metformin treatment group in a ratio of 1:1. Participants in each group will maintain their medication for 3 months after achieving the target blood glucose level and then stop it. These participants will be followed up for one year to determine remission rates in both groups.

**Discussion:**

In this study, we will establish that whether canagliflozin is superior to metformin in terms of remission rate in participants with newly diagnosed T2DM. The results of this trial may provide robust evidence regarding the efficacy and mechanisms of the action of sodium-glucose cotransporter-2 inhibitors (SGLT2is) in T2DM remission.

**Trial registration:**

ChiCTR2100043770(February 28, 2021).

**Supplementary Information:**

The online version contains supplementary material available at 10.1186/s12902-023-01461-9.

## Background

Increasing evidence has shown that it is possible to remit type 2 diabetes mellitus (T2DM) [1, 2]. Currently, three main methods are effective for remitting T2DM. Metabolic surgery is the most efficacious, with a maximum remission rate of approximately 70–80% at 1–2 years after surgery [3, 4], followed by lifestyle interventions with caloric restriction, with a 40–80% average remission rate at 1 year after intervention [5], and intensive insulin therapy, which can also provide remission in 30–70% of patients with T2DM [6, 7]. The most commonly used oral hypoglycemic agents for the treatment of T2DM have been understudied to date. There are relatively many studies on pioglitazone in remitting T2DM [8], and relevant studies have shown that pioglitazone can prolong the remission of T2DM, whereas gliclazide has a poor remission effect on T2DM [9]. Although metformin is the first-line drug for the treatment of T2DM, studies on its remission of T2DM are scarce, and only a few have published its use in combination with other drugs for T2DM remission [10]. A national diabetes cohort study from the United Kingdom reported that patients with a disease course of < 2 years and glycosylated hemoglobin A1c (HbA1c) level of < 7.0% had a greater probability of remission with metformin alone or no hypoglycemic drugs and a ≥ 10% reduction in body mass index (BMI) [11]. In addition, a study found that short-term intensive insulin therapy was not more effective than metformin therapy in newly diagnosed patients [12]. According to these studies, metformin is beneficial for remission in patients with newly diagnosed T2DM.

The mechanism through which metabolic surgery and lifestyle intervention initiate T2DM remission is mainly based on Professor Taylor’s “double vicious circle theory” [13]. Through weight loss, patients can remove excess fat load, reduce body fat content to below the “personal fat threshold”, improve liver and pancreas fat deposition, thus improving insulin resistance, and make de-differentiated islet cells re-differentiate into insulin-producing β-cells; taken together, all these effects lead to T2DM remission. Intensive insulin therapy alleviates T2DM by reducing blood glucose concentrations and improving glucose toxicity, lipotoxicity, proinsulin toxicity, proamylin toxicity, and cytokine toxicity. In recent years, new drugs have been used to treat diabetes clinically. Sodium-glucose cotransporter-2 inhibitors (SGLT2is) have attracted considerable attention because of their significant hypoglycemic effect and extensive clinical efficacy. Canagliflozin, a SGLT2i, is a new targeted oral glucose-lowering drug that inhibits the reabsorption of urine glucose sodium-dependent glucose transporter 2 in proximal renal tubular epithelial cells and increases the excretion of glucose in the urine. Specifically, it plays a hypoglycemic role through non-insulin-dependent mechanisms, thus, reducing blood glucose effectively and safely [14, 15]. In addition, SGLT2i improves insulin resistance and protects β-cell function [16]. This class of drugs not only reduces glucose levels but also causes weight loss, improves insulin resistance, and protects β-cell function while having multiple effects on the reversal and remission of T2DM [16]. Therefore, we hypothesized that canagliflozin could be effective for T2DM remission. Existing studies on the remission of T2DM have found that the shorter the course of the disease, the greater the probability of remission [17–19]; therefore, active intervention at the onset of diabetes is conducive to increasing the probability of remission. Based on this, we plan to select participants with a ≤ 3-year course of T2DM as the study subjects, and give them canagliflozin or metformin treatment to explore whether these two drugs are conducive to the remission of type 2 diabetes.

In recent years, the prevalence of diabetes has risen sharp [20, 21], which causes great physiological [22, 23], psychological [24, 25] and economic [21] harm to patients. After achieving the remission of diabetes through relevant measures, the life quality of patients can be improved, the incidence of complications and concomitant diseases can be reduced [26], the physical and mental health of patients is benefited, and the economic burden of families as well as society is reduced. Therefore, research and implementation of the remission of type 2 diabetes are particularly important, which are also the objectives of this study.

### Study objectives

The primary objectives are to assess the effects of canagliflozin and metformin on diabetes remission and determine whether canagliflozin is superior to metformin. The secondary objective is to explore the possible underlying mechanisms by which the two medications elicit T2DM remission.

## Methods and analysis

### Study design

**Fig. 1:**
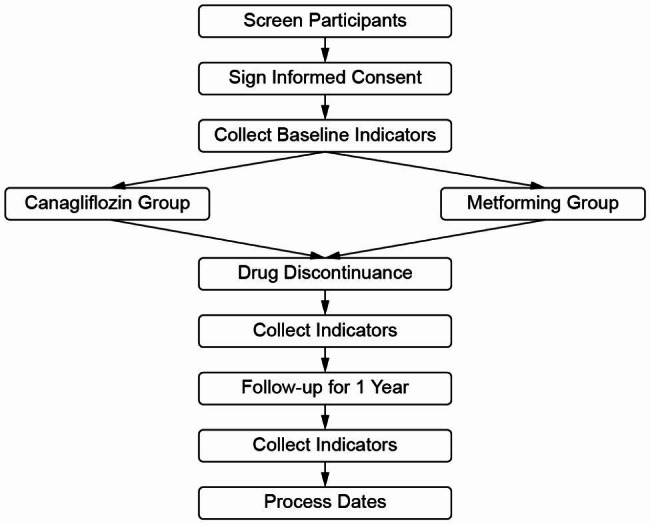
Participant flow diagram

This is a multicenter, parallel-group, randomized controlled, open-label trial (Fig. 1). The study has been approved by the Medical Ethics Committee of the Jiangsu Hospital of Integrated Traditional Chinese and Western Medicine and registered in the Chinese Clinical Trial Registry (ChiCTR2100043770). This study will be conducted at Jiangsu Hospital of Integrated Traditional Chinese and Western Medicine, Suzhou Hospital of Traditional Chinese Medicine, Suqian Hospital affiliated with Xuzhou Medical University, Xuzhou Mineral Group General Hospital, Xiamen Hospital of Traditional Chinese Medicine, and Nanjing Jiangning District People’s Hospital. A total of 184 participants will be enrolled and randomly assigned to the canagliflozin and metformin treatment groups.

### Participants

#### Inclusion criteria (during screening, all six criteria must be met)

1. 18–65 years old.
2. No limitation on sex.
3. Onset of T2DM (≤ 3-year course of the disease), diet and physical exercise alone for more than 8 weeks, and at least 8 weeks of no anti-diabetes medication (except short-term use for < 7 days).
4. 18.5 kg/m^2^ ≤ BMI ≤ 40 kg/m^2^.
5. 6.5% ≤ HbA1c ≤ 10%.
6. Able to understand the procedures and methods of this study, willing to complete the study in strict compliance with the clinical trial guidelines, and voluntarily signing informed consent.

#### Exclusion criteria (during screening, those who meet any of these criteria will be excluded)

(1) Severe diabetic complications include severe diabetic nephropathy (estimated glomerular filtration rate (eGFR) of < 60 mL/min/1.73 m^2^), diabetic foot (Wagner grade 2 or above) and diabetic retinopathy (with fundus hemorrhage affecting vision).

(2) Severe organ diseases or other severe primary diseases such as liver and kidney dysfunction, heart failure, cerebral infarction, and malignant tumors.

(3) Severe cognitive dysfunction and unclear language expressions.

(4) Mental illness.

(5) Severe chronic infection.

(6) History of acquired immunodeficiency syndrome and drug abuse.

(7) Pregnant or lactating women.

(8) Participation in another clinical trial.

(9) Blood donation or blood loss of ≥ 400 mL within 4 weeks before screening or recipient of blood transfusion.

(10) Fasting plasma/serum glucose > 15 mmol/L.

(11) Alanine aminotransferase ≥ 2.5 × upper limit of normal(ULN).

(12) Total bilirubin ≥ 1.5 × ULN.

(13) Hemoglobin ≤ 100 g/L and neutrophils < 1.5 × 10^9^/L.

(14) Renal injury (eGFR < 60 mL/min/1.73 m^2^ or serum creatinine ≥ 2.5 × ULN or on kidney dialysis).

(15) Blood thyroid-stimulating hormone level beyond the normal range and has clinical significance, as assessed by researchers.

(16) Parathyroid hormone > 1.5 × ULN.

(17) Myocardial enzyme spectrum (creatine kinase and creatine kinase isoenzyme ck-mb) > 3 × ULN.

(18) Fasting triglyceride ≥ 5.64 mmol/L (500 mg/dL).

(19) Blood amylase and lipase levels exceed the upper limit of the normal range and are clinically significant, according to the researchers’ assessment.

(20) Other factors and laboratory outliers of clinical significance that the investigators believe may interfere with interpreting the efficacy and safety data in this study.

#### Exit and withdrawal criteria

(1) Pregnant women or women planning for pregnancy.

(2) Intolerable adverse events.

(3) Worsening of blood glucose levels.

(4) Severe or repeated hypoglycemia.

(5) Pancreatitis.

(6) Participants who do not take medication nor accept testing as required.

(7) Participants who cannot complete the study as planned or have other factors that may affect the efficacy or safety evaluation.

(8) Participants who participate in other clinical trials during the trial.

(9) Participants who voluntarily withdraw from the study with informed consent.

(10) Participants who do not explicitly raise the request to withdraw from the test but no longer accept medication, testing, and follow-up.

### Participant recruitment

The recruitment period of this study will start in December 2023. Participants will be invited to participate in the study through meetings, electronic and social media. The recruitment will end on 31 December 2025.

### Randomisation and interventions

A total of 184 participants will be randomly allocated to receive either canagliflozin or metformin treatment (1:1). The randomization operation will be generated by an independent statistician, who will ensure the balance of participants according to age, sex, HbA1c and BMI and course of their disease when grouping, and a code list will be directly allocated to the main investigators of every center. The intervention content of the two groups is as follows.

1. Canagliflozin treatment group: For oral treatment, the participants will receive canagliflozin tablets (Janssen-Cilag S.p.A., Ltd., National Drug Approval: H20170375) once daily, 100–300 mg per time, with a minimum dose of 100 mg per day and maximum dose of 300 mg per day.
2. Metformin treatment group: For oral treatment, the participants will receive metformin hydrochloride tablets (Sino-American Shanghai Squibb Pharmaceutical Co., Ltd., National Drug Approval: H20023370 (0.5 g × 20 tablets)/Sino-American Shanghai Squibb Pharmaceutical Co., Ltd., National Drug Approval: H20023371 (0.85 g × 10 tablets × 2 plates)), 1–3 times a day, 500–2000 mg per time, with a minimum dose of 500 mg per day and maximum dose of 2550 mg per day.

Regarding weekly titration for oral drug metering, the titration targets are fasting blood glucose (FBG) (3.9–5.6 mmol/L) and blood glucose at 2 h after each of the three meals (5.0–7.0 mmol/L). After meeting the above criteria, the participants will continue the medication for 3 months and then stop.

**Fig. 2:**
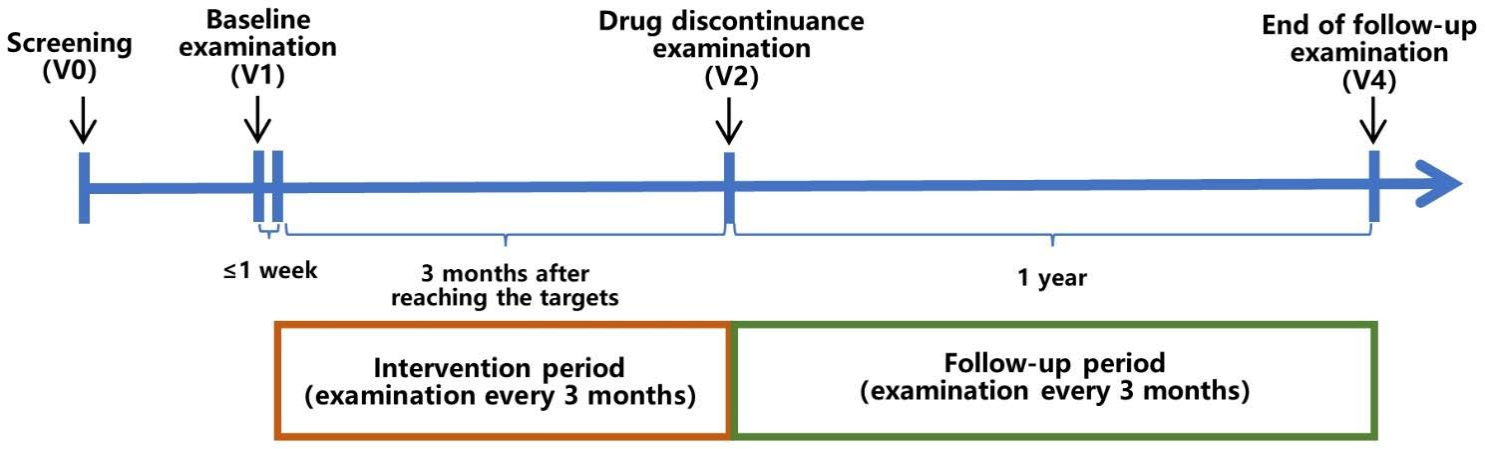
Study timeline

Routine diabetes health education will be provided to all participants during the treatment period. Routine diet control and appropriate exercises will be planned. Follow-up will be conducted monthly for the first 3 months after the end of treatment and every 3 months thereafter for 1 year (Table 1; Fig. 2). Participants with FBG > 7.0 mmol/L and postprandial blood glucose (PBG) > 10 mmol/L during the follow-up period are defined as having hyperglycemia recurrence, which will be treated according to the Chinese guidelines for the diagnosis and treatment of T2DM and is defined as not remitted.

**Table 1:** Participant visit schedule

**Research Stage**	**Screening**	**Baseline**	**Intervention Period**				**Follow-up Period**		
			**Start of treatment**	**Dose titration period** **(weekly)**	**Maintenance treatment** **(monthly)**	**Drug discontinuance**	**First three months** **(monthly)**	**Last nine months** **(every three months)**	**End of the follow-up**
Informed consent		**×**	**×**	**×**	**×**	**×**	**×**	**×**	**×**
Participant information		**×**	**×**	**×**	**×**	**×**	**×**	**×**	**×**
Medical history		**×**	**×**	**×**	**×**	**×**	**×**	**×**	
Clinical examination		**×**	**×**	**×**	**×**	**×**	**×**	**×**	
Inclusion and exclusion criteria		**×**	**×**	**×**	**×**	**×**	**×**	**×**	**×**
Assignment filter number		**×**	**×**	**×**	**×**	**×**	**×**	**×**	**×**
Weight, height, and BMI									
Body composition	**×**		**×**	**×**	**×**		**×**	**×**	
Waist circumference									
Blood pressure and resting heart rate									
HbA1c, FPG, 2hPG			**×**	**×**	**×**		**×**		
FCP, 2hCP, FINS, INS-2 h	**×**		**×**	**×**	**×**		**×**		
Blood biochemistry, blood routine			**×**	**×**	**×**		**×**		
Thyroid function		**×**	**×**	**×**	**×**	**×**	**×**	**×**	**×**
Routine urine, urine microalbumin			**×**	**×**	**×**		**×**		
ECG, UCG		**×**	**×**	**×**	**×**	**×**	**×**	**×**	**×**
Abdominal ultrasonography	**×**		**×**	**×**	**×**	**×**	**×**	**×**	
Eye-ground photography, electromyography, color doppler ultrasound of blood vessels of the neck and lower limbs	**×**		**×**	**×**	**×**	**×**	**×**	**×**	
Stored samples (blood and stool)	**×**		**×**	**×**	**×**		**×**	**×**	
DSQLS、SDSCA	**×**		**×**	**×**	**×**		**×**	**×**	
Medication, diet, and exercise guidance	**×**								
Treatment of comorbidity	**×**								
Blood glucose monitoring	**×**	**×**							
Dietary and sport record	**×**	**×**							
Occurrence of hypoglycemia, ketosis, and other adverse reactions	**×**	**×**							
Distribute participant diaries		**×**	**×**	**×**	**×**	**×**	**×**	**×**	
Recall and review participant diaries	**×**	**×**	**×**	**×**	**×**	**×**	**×**	**×**	

BMI, body mass index; ECG, electrocardiograph; FCP, fasting C-peptide; FINS, fasting serum insulin; HbA1c, glycosylated hemoglobin A1c; 2hCP, 2 - hour postprandial C-peptide; INS-2 h, 2 - hour postprandial insulin; DSQLS, diabetes specific quality of life scale; SDSCA, summary of diabetes self care activities; UCG, ultrasonic cardiogram

### Outcomes

#### Primary outcome

Remission rates.

This is the percentage of participants in each group who meet the remission criteria during follow-up: HbA1c < 6.5% maintained for > 3 months after the treatment discontinuation [27].

#### Secondary outcomes

1. General physical characteristics, including weight, height, BMI, abdominal circumference, body composition, heart rate, and blood pressure.
2. Blood glucose-related indexes: HbA1c, FBG, PBG, fasting insulin, fasting C-peptide, 2-h postprandial insulin, and 2-h postprandial c-peptide levels; homeostasis model assessment of insulin resistance; and homeostasis model assessment of β-cell function.
3. Related indexes of lipid metabolism: plasma levels of triglycerides, total cholesterol, low-density lipoprotein cholesterol, and high-density lipoprotein cholesterol.
4. Complications-related indicators: eye-ground photography, electromyography, and color doppler ultrasound of blood vessels in the neck and lower limbs.
5. Visceral fat content: abdominal ultrasonography.
6. Intestinal flora.
7. Metabolomics.

#### Safety outcomes

Participants will be followed up by telephone every month to inquire about their adverse reactions and events, and physical examinations, laboratory examinations as well as auxiliary detections will be conducted every three months. If there is a problem, take appropriate solutions and treatment measures, and record the situation in time.

Monitoring indexs: clinical symptoms; vital signs; electrocardiogram; blood and urine routine tests; liver and kidney functions; electrolyte levels; urinary albumin/creatinine ratio; myocardial enzyme profile; blood amylase level; thyroid function; eGFR; and blood/urine pregnancy test; and incidence of hypoglycemia, ketosis hypoglycemia and cardiovascular and cerebrovascular events.

#### Life quality outcomes

Diabetes specific quality of life scale (DSQLS) and summary of diabetes self care activities (SDSCA) will be used to assess life quality outcomes.

#### Compliance outcomes

The compliance outcomes include the implementation rates of various examinations, medications, and diet, compliance with exercise, return visit rate, and follow-up evaluation.

### Visits and measurements

#### Follow-up visit

At the beginning of treatment, participants will be required to visit the centre every week to report their blood glucose monitoring status, and receive adjustment of the dosage and frequency to achieve a fast and stable blood glucose standard. When the participant’s blood glucose level reaches the standard, the visit will be repeated every 2 ~ 4 weeks until the end of the treatment period. After drug withdrawal, participants will be followed up by telephone every month and return to the centre every 3 months until the end of follow-up.The specific follow-up items are shown in Table 1.

#### Anthropometric measures

Anthropometric measurements will be performed at each research center by trained surveyors using standardized operating procedures. The participants will be instructed to remove their shoes and heavy clothes when measuring their weight; height and waist circumference will be measured in centimeters using a unified measuring instrument. The attending physicians will ask and record the participants’ clinical symptoms, perform physical examinations, and guide them to complete the DSQLS and SDSCA.

#### Biochemical measures

Participants will be required to fast at least 10 h before the study visit and return to the center between 6 and 10 am. They will be instructed to stop smoking and abstain from caffeine and alcohol 10 h before the study visit to ensure that all fasting blood samples are collected before taking medication in the morning. Biochemical tests, such as hematuria, will be completed at each center, and stool and blood samples of each participant will be collected and sent to professional institutions for the subsequent intestinal flora and metabolomics testing.

#### Participant diary

Each participant will be provided a participant diary and instructed to record blood glucose, blood pressure, weight, abdominal circumference, medication, diet, exercise, and adverse reactions, such as hypoglycemia. We will check and record the participant’s diary during each follow-up and return visit.

### Sample size calculation

This is a controlled trial aims at exploring the remission effect of canagliflozin on T2DM. The experimental group is the canagliflozin group, and the control group is the metformin group. The main observation index is the remission rate. We referred to relevant studies and set the remission rate for metformin as 20.9%, similarly the estimated remission rate of canagliflozin was also 20.9%. For non-inferiority design, the cut-off value was 2%, α = 0.025, and power = 0.80. The sample size calculated by PASS 15 software was 73 cases in each group, and at least 92 participants should be enrolled in each group at baseline, for a total of 184 participants.

### Data collection

All participants’ information will be collected and sorted by the full-time personnel in each center. After sorting, they will be checked by another staff member. After confirmation, the information will be uploaded to the Metabolic Management Center for timely storage to ensure the continuity and accuracy of participants’ inspection data. A confidentiality agreement shall be signed with the Metabolic Management Center, and each center shall appoint a network docking contact person responsible for data security assurance, storage, and export. The export of data shall be approved by the main researchers.

### Data analysis

All statistical analyses will be performed by full-time statisticians using R Studio (Version 1.2.5001, R Studio Inc., Boston, MA, USA). The normality test will be used to determine whether the data are normally distributed. Continuous variables with a normal distribution will be reported as the mean ± SD, and ordinal and non-normally distributed variables will be reported as the median and interquartile range. Independent samples t-tests or Mann-Whitney U-tests will be used for comparisons, as appropriate. Categorical variables will be reported as counts and proportions, and the Pearson χ^2^ test or Fisher’s exact test will be used for comparisons, as appropriate.

The primary outcomes (remission rate 3 months after drug withdrawal and after 1 year of follow-up) will be compared between groups using Pearson’s χ^2^ test or Fisher’s exact test. Logistic regression will be used to adjust for potential confounders. Variables with P < 0.05 in the univariate comparisons, and known confounders that could impact the efficacy, will be included in the multivariable logistic regression models.

Subgroup analyses will be conducted of the primary outcomes stratified by age group (young, 18 ~ 35 years; middle-aged, 36 ~ 59 years; older, 60 ~ 65 years); duration of disease (<1 year, 1 ~ 2 years, 2 ~ 3 years); BMI (18.5 ~ 27.9 kg/m^2^, 28 ~ 40 kg/m^2^); and sex, using the same methods of analysis.

For the secondary outcomes, continuous data will be analyzed using the linear mixed effects model, which will use the random intercept model to explain the internal correlation of participants. Discontinuous data will be analyzed similar, but the generalized linear model appropriate to the distribution assumptions and link functions will be used. The analysis of all secondary outcome indicators also corrected for potential confounders (including variables with P < 0.05 in the univariate comparisons and known confounders that could have an impact on the efficacy). The safety indicators will be analyzed using the same methods used to analyze the main outcome indicators.

Missing values will be filled using multiple imputation methods, and sensitivity analyses will be performed.

Two-tailed P-values will be used, and P-values < 0.05 will be regarded as statistically significant. Point estimates will be reported with 95% confidence intervals.

### Data verification

The research coordination center will be responsible for data verification, including verification of missing data, untrue values, cross-checking of inconsistent data, and spot-checking of data. All data problems will be fed back to the research center for timely processing. Data inspectors will generate online data challenge reports summarizing the number and types of research data challenges. Researchers in the research centers are responsible for the timely review and resolution of data problems.

### Quality control report

The quality control committee will use quality assessment indicators to record data quality and provide feedback to each research center, continuously track these quality assessment indicators in quality control reports, generate quality control reports, and distribute them to each research center. It will also conduct regular telephone contacts or follow-up discussions to ensure the research quality of each research center.

### Ethical design and trial registration

This study has been approved by the Medical Ethics Committee of the Jiangsu Hospital of Integrated Traditional Chinese and Western Medicine (2021-LWKYZ-002) according to the Medical Research Involving Human Subjects Act and the standards of Good Clinical Practice and registered in the Chinese Clinical Trial Registry (ChiCTR2100043770) and will be conducted in accordance with the Declaration of Helsinki.

## Discussion

In the 1960s, researchers began to study diabetes remission [28]. Simultaneously, they explored and researched for decades with considerable results [6]. Lifestyle interventions and metabolic surgery can remit diabetes by improving insulin sensitivity and preserving islet β-cell function through weight loss, whereas insulin-intensive therapy can remit diabetes by protecting only the islet β-cell function. These three methods have good remission effects [19]; however, patients receiving diet therapy are usually given a low-calorie diet or even a very low-calorie diet. A strong sense of hunger caused by this diet may make it difficult for patients to continue the diet. After the restriction ends, if the patient cannot continue diet control, they may regain weight, consequently affecting disease remission [29]. Bariatric surgery has prescribed surgical indications and is unsuitable for all patients with T2DM [30]. Intensive insulin therapy is difficult to administer and leads to poor patient compliance; insulin therapy is also associated with an increased risk of weight gain [31]. In addition, studies on the remission of T2DM with oral hypoglycemic drugs are fewer than those with other methods [32, 33]. Further, the results of previous studies have shown that the remission effects of various drugs are inconsistent and not ideal.

In this study, we believed that oral hypoglycemic drugs for the remission of incipient T2DM could improve the remission of primary T2DM. Also, the findings obtained could promote further studies on SGLT2is. Improving the remission rate of primary diabetes can reduce the treatment cost of T2DM and the burden on society and family, which is significant for preventing and treating T2DM.

### Electronic supplementary material

Below is the link to the electronic supplementary material.

Supplementary Material 1

## Data Availability

The original contributions presented in the study are included in the article/supplementary material. Further inquiries can be directed to the corresponding authors.

## Abbreviations

BMI: Body mass index
DSQLS: Diabetes specific quality of life scale
ECG: Electrocardiograph
eGFR: Estimated glomerular filtration rate
FBG: Fasting blood glucose
FCP: Fasting C-peptide
FINS: Fasting serum insulin
HbA1c: Hemoglobin A1c
PBG: Postprandial blood glucose
SDSCA: Summary of diabetes self care activities
SGLT2is: Sodium-glucose cotransporter-2 inhibitors
2hCP: 2 - hour postprandial C-peptide
INS-2h: 2 - hour postprandial insulin
T2DM: Type 2 diabetes
ULN: Upper limit of normal
